# Supplementary material for: Content-rich biological network constructed by mining PubMed abstracts
Source: BMC Bioinformatics. 2004 Oct 8;5:147. doi: 10.1186/1471-2105-5-147 (PMC528731; doi:10.1186/1471-2105-5-147)
Supplement: Additional File 2 — The original results of the above study (non-essential files are deleted to keep the file size under the limit set by BMC bioinformatics). [file 1471-2105-5-147-S2.bz2 › chilibotAdditionalFile2/dip20/681ID11493686E17996/html/DNAB_TUS.html]

 


 **DNAB** and **TUS** 
  
Found 11 abstracts in PubMed, retrieved 11.  
 

 What does Google say? 
 PDF only 
| .edu only 

---

**Interactive relationship** (e.g. stimulation, inhibition, etc)

**Neutral relationship**- We show further that replication termination involves protein protein interaction between  **Tus**  and  **DnaB**  at a critical region of  **Tus**  protein, called the L1 loop.  Ref: 11493686 Proc Natl Acad Sci U S A, 2001
- Several mutations located in the L1 loop region not only reduced the protein protein interaction but also eliminated or reduced the ability of the mutant forms of  **Tus**  to arrest  **DnaB**  at a Ter  [ **TUS** ]  site.  Ref: 11493686 Proc Natl Acad Sci U S A, 2001
- Using yeast forward and reverse two hybrid analyses, we have discovered that the replication terminator protein  **Tus**  of Escherichia coli physically interacts with  **DnaB**  helicase in vivo.  Ref: 11493686 Proc Natl Acad Sci U S A, 2001
- They appeared to bind Ter  [ **TUS** ]  DNA normally but were ineffective in fork arrest as ascertained by in vitro Escherichia coli  **DnaB**  helicase and replication assays.  Ref: 10064700 J Mol Biol, 1999
- A173T had a 4100 fold lower affinity for Ter  [ **TUS** ]  sites than wild type  **Tus**  and was unable to halt DNA replication in vivo or inhibit  **DnaB**  catalyzed strand displacement in an in vitro helicase assay.  Ref: 8051142 J Biol Chem, 1994

**Non-interactive relationship** (e.g. studied together, co-existance, homology, etc.)

- In order to probe the molecular mechanism of this inhibition, we have used a strand displacement assay to investigate the effect of  **Tus**  on the DNA helicase activities of  **DnaB** , PriA, UvrD helicase II, and the phi X type primosome.  Ref: 1317865 J Biol Chem, 1992
- Using the ter  [ **TUS** ]  protein and highly purified  **dnaB**  helicase, we show that the terminator protein is a DNA sequence specific contra helicase, i.e., the protein when bound to its recognition sequence tau strongly impedes the ATP dependent unwinding of double stranded DNA.  Ref: 2684415 Cell, 1989
- At least one mutation, E49K, significantly reduced  **Tus**   **DnaB**  interaction and almost completely eliminated the contrahelicase activity of  **Tus**  protein in vitro without significantly reducing the affinity of the mutant form of  **Tus**  for Ter  [ **TUS** ]  DNA, in comparison with the wild type protein.  Ref: 11493686 Proc Natl Acad Sci U S A, 2001
- When the substrate was a short oligomer hybridized to a circular single stranded DNA, strand displacement by  **DnaB** , PriA, and the primosome in both directions, but not UvrD, was blocked by  **Tus**  in a polar fashion.  Ref: 1317865 J Biol Chem, 1992
- In addition, we found that A173V was a weak inhibitor of  **DnaB**  catalyzed strand displacement in vitro, yet halted DNA replication in vivo at 75% of the efficiency of wild type  **Tus** .  Ref: 8051142 J Biol Chem, 1994
- The 27 kd protein also neutralizes the contrahelicase activity of ter  [ **TUS** ]  protein, allowing  **dnaB**  helicase to unwind DNA past a tau ter  [ **TUS** ]  complex.  Ref: 8380756 Cell, 1993
- The target for blockage within the replication complex can now be ascribed to the inability of  **dnaB**  helicase to separate the duplex strands when it encounters ter  [ **TUS** ]  bound by TBP.  Ref: 2556700 Proc Natl Acad Sci U S A, 1989
- Other helicases, such as rep and uvrD proteins, that translocate on DNA and displace strands in the direction opposite to that of  **dnaB**  protein are also blocked, but only when the TBP bound ter  [ **TUS** ]  is oriented in the other direction.  Ref: 2556700 Proc Natl Acad Sci U S A, 1989
